# Treatment of Inoperable Cancer

**Published:** 1915-07

**Authors:** 


### ABSTRACTS

---

**Treatment of Inoperable Cancer.** Silas P. Beebe, New York. The therapeutic agent is due to Alexander Horovitz, Ph. D. Doctor Beebe and Dr. J. Wallace Beveridge, working in conjunction with Dr. Horovitz, after many months of clinical experimentation, have been able to use the product as an extract administered subcutaneously. ''At the point of injection in normal tissue an active local reaction is produced; this reaction is evidenced by swelling, redness, heat, and tenderness. Then follows a general leucocytosis with a relatively high lymphocytosis, some rise in temperature, and occasionally, a chill of varying intensity and duration. Now if the local area which receives the injection is examined microscopically, there are found all the characteristics of a moderately acute inflammatory reaction with a relatively large leucocytic infiltration. When such extracts were injected directly into a transplantable rat sarcoma, the characteristic reaction followed and was accompanied by a peculiar necrosis of the tumor cells with complete regression.

When the skin over the tumor, prior to the injection, is ulcerated, the affected area rapidly degenerates and a mass of necrotic tissue is discharged, followed by healing, while if the skin is not broken or ulcerated, the reaction following the injection produced a marked infiltration of serum and leucocytes, particularly around the borders of the tumor, the tumor itself was gradually absorbed, and there was an apparent complete restoration of normal cellular conditions.''

Subcutaneous injections of this extract have to a considerable extent displaced the direct tumor injection, having the obvious advantage of permitting a more certain dose, of bringing this therapeutic agent directly in contact with the growing border of the malignant cells, and producing in the depths of the tumor rather than on its surface an intense reaction, which appears to be unfavorable for the continued growth of the tumor. When these injections were first begun in human subjects, they were always confined to the growth itself. More recently they have been given subcutaneously in the arm, and it has been interesting to note that when so given there has been observed fairly definite reactive responses in the growth; these reactions in the growth are evidenced by swelling, temporary increase in pain, followed a few hours later by a considerable relief from pain, and in some forms of tumor by softening of the growth and a gradual diminution in its size.

Doctor Beebe's paper contains a report of two groups of cases, the first, in which X-ray from a Coolidge tube formed a part of the treatment which was not so successful and satisfactory as in the second group, treated entirely by hypodermic injection of the extract, no other therapeutic measures being used. The following two cases are from Dr. Beebe's preliminary report:

Case XI. Man, aged fifty years, had recurrent colloid carcinoma of the rectum. Kraske operation two years ago. In July recurrence became troublesome, and at the time of his admission to the hospital there was extensive involvement of the tissues, in and about the rectum and including the base of the bladder. Patient had severe pain, great difficulty in defecating. It was impossible at the time of this admission to pass a rectal tube, and the bladder irritation was so severe as to cause almost constant tenesmus. Injections at the hospital were made directly into the growths. During his stay in the hospital of six weeks, sixteen injections were made; following the earlier ones, there was marked reaction accompanied by rise of temperature and an occasionel chill. X-ray examination revealed involvement of sacrum. Large broken down masses of tumor were discharged. Pain and irritation about the base of the bladder gradually diminished. The swelling and pain about the sacrum entirely disappeared, the tumor masses in the rectum were in part absorbed and in part broken down, and were discharged. At the end of six weeks the patient left the hospital, free from pain, with normal control of bladder. Patient was passing formed stools, the rectum admitted forefinger easily without pain, and tumor masses could not be felt. After leaving the hospital, the patient had a few injections in the arm, he continued to gain in weight and strength, and his general condition continued to improve.

Case XV. Woman, aged fifty-one years, had a recurrent inoperable carcinoma of the breast. Recurrence involved the fifth and sixth rib, at the lower point of the old scar. Mass about three inches in diameter, very hard, the surface red. Some exudate appeared at the apex of the growth. Patient had intense pain along the left arm and shoulder, including the left side of the neck. Left arm markedly edematous and painful to touch. Edema extended to the region above the clavicle. Patient had had X-ray treatment for a short time prior to admission to hospital without effect. Treatment was entirely by injection into the tumor and into the arm on the right side. At the time of her admission and previously, patient had temperature of 100° to 101° F., which finally became normal. During the period of twenty-six days at the hospital, patient received eighteen injections, two being in the right arm. After her discharge, patient continued to receive the injections at weekly intervals into the right arm. When the patient left the hospital the induration had entirely disappeared, edema in the arm and above the clavicle had been absorbed, and she had no pain. The central portion of the tumor mass was marked by a scab, about a quarter of an inch in diameter, representing a point of an old sinus through which most of the tumor mass had been discharged. After she left the hospital, this area entirely healed, the patient returned to work and was subsequently entirely well.

Prof. Beebe, in summing up his observation states:—"In spite of the somewhat complex and unusual character of the remedy employed, the evidence warrants further use of this method of treatment. In judging the merit of a treatment for inoperable cancer, it is probably wise to discount such matters as the relief from pain and the improvement of the general physical condition, because, while these matters are of much concern to the patient, they are to a considerable degree subjective in character and may to some extent be expected to follow the employment of any new method which stimulates the patient with faith and hope."

